# Vacuum sealing drainage to treat Fournier’s gangrene

**DOI:** 10.1186/s12893-023-02109-0

**Published:** 2023-07-26

**Authors:** Ju-hua Chen, Yu-bo Li, De-gang Li, Xiao-mei Zeng, Qiu-yuan Yao, Jun Fu, Gong-he Wang, Xiao-yan Huang

**Affiliations:** 1 Colorectal Surgery Division, Affiliated Hospital of Jinggangshan University, Ji’an, 343000 Jiangxi China; 2 Colorectal Surgery Division, The First Affiliated Hospital of Guangxi University of Chinese Medicine, Dongge Road 89-9, Nanning, 530023 Guangxi China; 3 Colorectal Surgery Division, Traditional Chinese Medicine of Guiping city, Guiping, 537200 Guangxi China

**Keywords:** Fournier’s gangrene, Vacuum sealing drainage, Debridement, Wound reconstruction, Soft tissue infection, Necrosis

## Abstract

**Background:**

Vacuum sealing drainage (VSD) is widely applied in complex wound repair. We aimed to compare traditional debridement and drainage and VSD in treating Fournier’s gangrene (FG).

**Methods:**

Data of patients surgically treated for FG were retrospectively analyzed.

**Results:**

Of the 36 patients (men: 31, women: 5; mean age: 53.5 ± 11.3 [range: 28–74] years) included in the study, no patients died. Between-group differences regarding sex, age, BMI, time from first debridement to wound healing, number of debridements, FGSI, and shock were not statistically significant (*P* > 0.05). However, lesion diameter, colostomy, VAS score, dressing changes, analgesic use, length of hospital stay, and wound reconstruction method (χ^2^ = 5.43, *P* = 0.04) exhibited statistically significant differences. Tension-relieving sutures (6 vs. 21) and flap transfer (4 vs. 2) were applied in Groups I and II, respectively.

**Conclusion:**

VSD can reduce postoperative dressing changes and analgesic use, and shrunk the wound area, thereby reducing flap transfer in wound reconstruction.

## Introduction

**Fig. 1:**
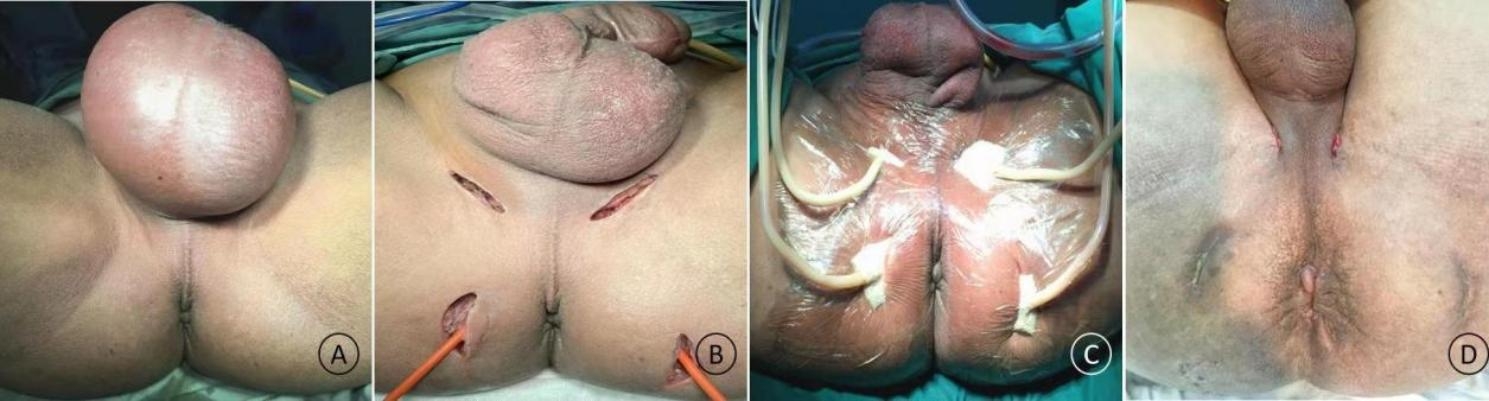
A 56-year-old man with FG **(A)** had lesions involving the perianal, perineal, and scrotal regions accompanied with skin necrosis. **(B)** Drainage was performed intraoperatively through four small incisions made away from the anus. **(C)** VSD sponges were inserted through the incisions and vacuum suction was maintained postoperatively. The patient was discharged after a single VSD session. **(D)** Re-examination at 25 days postoperatively showed that the perianal region was in good condition

**Fig. 2:**
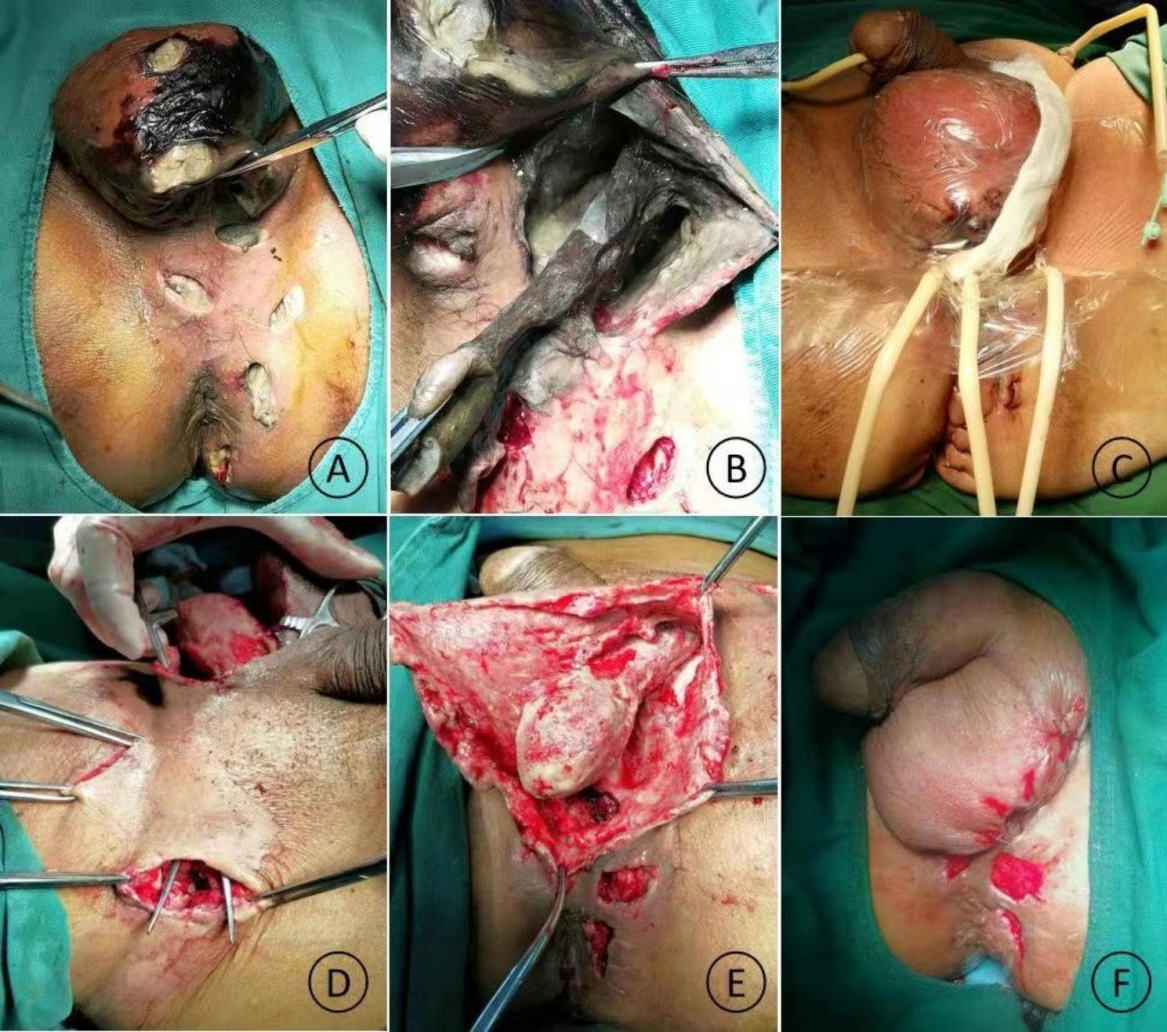
A 66-year-old man with FG **(A)** had lesions involving the perianal, perineal, scrotal, and left inguinal regions accompanied with scrotal skin necrosis. **(B)** Thorough surgical removal of necrotic tissue was performed, **(C)** VSD sponges were inserted into the wound, and vacuum suction was maintained postoperatively. **(D, E)** The growth of fresh granulation was observed after one session of VSD). **(F)** Wound construction using tension-relieving sutures was performed after three VSD sessions

Fournier’s gangrene (FG) is a severe form of soft tissue infection that occurs in the perianal, perineal, and genital regions characterized by purulent necrosis caused by multiple types of anaerobic and aerobic microorganisms (Figs. 1 and 2). FG causes septic shock and multiple organ failure with mortality rates ranging from 4 to 67% [1, 2]. Surgical debridement removes necrotic tissue and drainage of lesions facilitates rapid control of systemic infection. However, FG causes extensive infection and requires repeated debridement, which leads to enlarged surgical wounds that make subsequent wound reconstruction difficult.

A number of treatments are currently available for postoperative wound care in patients with FG, including hyperbaric oxygen therapy, application of unprocessed honey, growth factors and vacuum-dressing technologies [3]. Vacuum-assisted closure (VAC) is a vacuum-dressing technique that reduces the frequency of postoperative dressing changes, alleviates pain, decreases analgesic use and increases postoperative comfort in patients with FG [3–8]. Another vacuum-dressing technique known as vacuum sealing drainage (VSD) is similar to VAC, but application and drainage effects differ due to different material designs. To our knowledge, the use of VSD to treat FG has not been reported. We compare the clinical effects achieved using VSD with those of traditional debridement and drainage in FG treatment.

## Materials and methods

### General data

We retrospectively analyzed the clinical information of 36 patients with FG who were admitted to our hospital between August 2013 and April 2022. The patients were divided into the traditional debridement and drainage (Group I) and VSD (Group II) groups. All patients provided written consent before receiving surgical treatment, and those with necrotizing fasciitis secondary to rectal tumors or other sites were excluded. FG was diagnosed based on medical history, clinical presentation, and physical examination. Patients primarily presented with redness and swelling of the skin in the perianal, perineal, and genital regions, which was accompanied in some cases by erythema, blisters, and crepitus, or even skin ulcerations, gangrene, and foul-smelling purulent discharge. The following clinical data were collected: sex, age, smoking history, alcohol-drinking history, body mass index (BMI), lesion diameter, length of hospital stay (LOS), visual analogue scale (VAS) score, frequency of dressing changes per day, frequency of analgesic use per day, time from first debridement to wound healing, occurrence of shock, receipt of intensive care unit (ICU) treatment, number of debridements, receipt of colostomy, predisposing diseases, Fournier’s gangrene severity index (FGSI), wound reconstruction method, and mortality.

### Treatment

All patients were immediately administered fluid resuscitation, anti-infection treatment using broad-spectrum antibiotics, and preoperative preparations upon hospital admission, and surgical debridement was performed on the day of admission. Intrathecal anesthesia was the preferred method, but general anesthesia with endotracheal intubation was adopted in patients for whom intrathecal anesthesia was contraindicated or those who required colostomy.

Group I: Intraoperative removal of necrotic tissue was thoroughly performed until healthy tissue appeared. Upon completion of debridement, the wound was alternately rinsed with hydrogen peroxide and saline and subsequently covered with an iodophor-impregnated dressing. Depending on the intraoperative status of the patients, a rubber drainage catheter or loose rubber band seton was placed when required. Postoperative wound care was also performed using an iodophor-impregnated dressing, with dressing changes at least twice daily. When postoperative increase in necrotic tissue or lesion spread was observed, repeat debridement was performed in the operating room.

Group II: In the absence of necrosis in the skin of infected lesions, a small incision (d ≈ 3 cm based on our experience) was usually made in the perineal region, and the surgeon’s index finger was inserted into the incision to adequately separate the infected lesions. The great extent of infection usually required multiple small incisions in the perianal and perineal regions (Fig. 1) to enable interconnection of subcutaneous infected lesions between the incisions for contra-aperture drainage. Necrotic tissue beneath the skin was subsequently removed along the surgical incisions. In cases with skin necrosis of the infected lesions, the necrotic skin tissue was completely removed, with care taken to avoid or minimize incision of non-necrotic skin tissue, to keep the wound as small as possible. The above steps were repeated along the incision to achieve complete removal of necrotic tissue and to establish contra-aperture drainage (Fig. 2). Upon completion of debridement, the wound was alternately rinsed with hydrogen peroxide and saline to prevent active bleeding from the wound. VSD sponges were subsequently cut to appropriate sizes based on the incision size and depth and separately inserted through various incisions to enable adequate lesion drainage and wound coverage. Last, the wound was sealed using a semi-permeable film dressing. Central vacuum suction was maintained postoperatively at pressures of -200 to -120 mm Hg (1 mm Hg = 0.133 kPa).Daily postoperative rinsing was performed using hydrogen peroxide (5 mL) and saline (100 mL) at the side holes of the drainage catheter to prevent catheter blockage. The VSD dressing was immediately changed if a closed environment could not be maintained due to blockage of the drainage catheter or film detachment; otherwise, VSD dressing changes were performed once every three to five days.

### Statistical analysis

Data were statistically analyzed using IBM SPSS 21.0 (IBM SPSS Version 21.0. Armonk, NY). Categorical variables were compared using the χ² test or Fisher’s exact test, and continuous variables were compared using the *t-*test or analysis of variance (ANOVA). Statistical significance was set at *P* < 0.05.

## Results

**Fig. 3:**
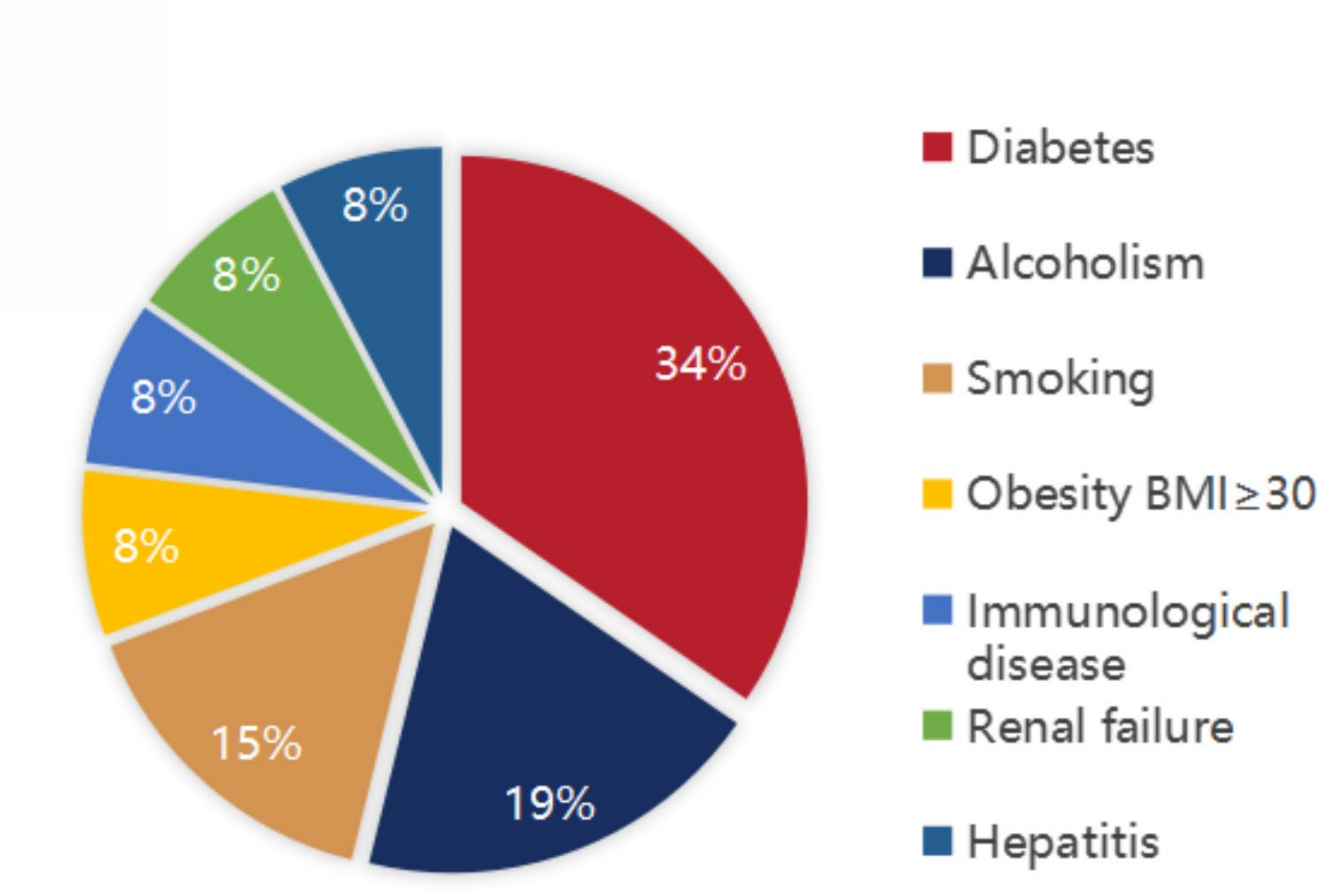
Proportions of predisposing diseases in patients with FG (n = 36)

**Fig. 4:**
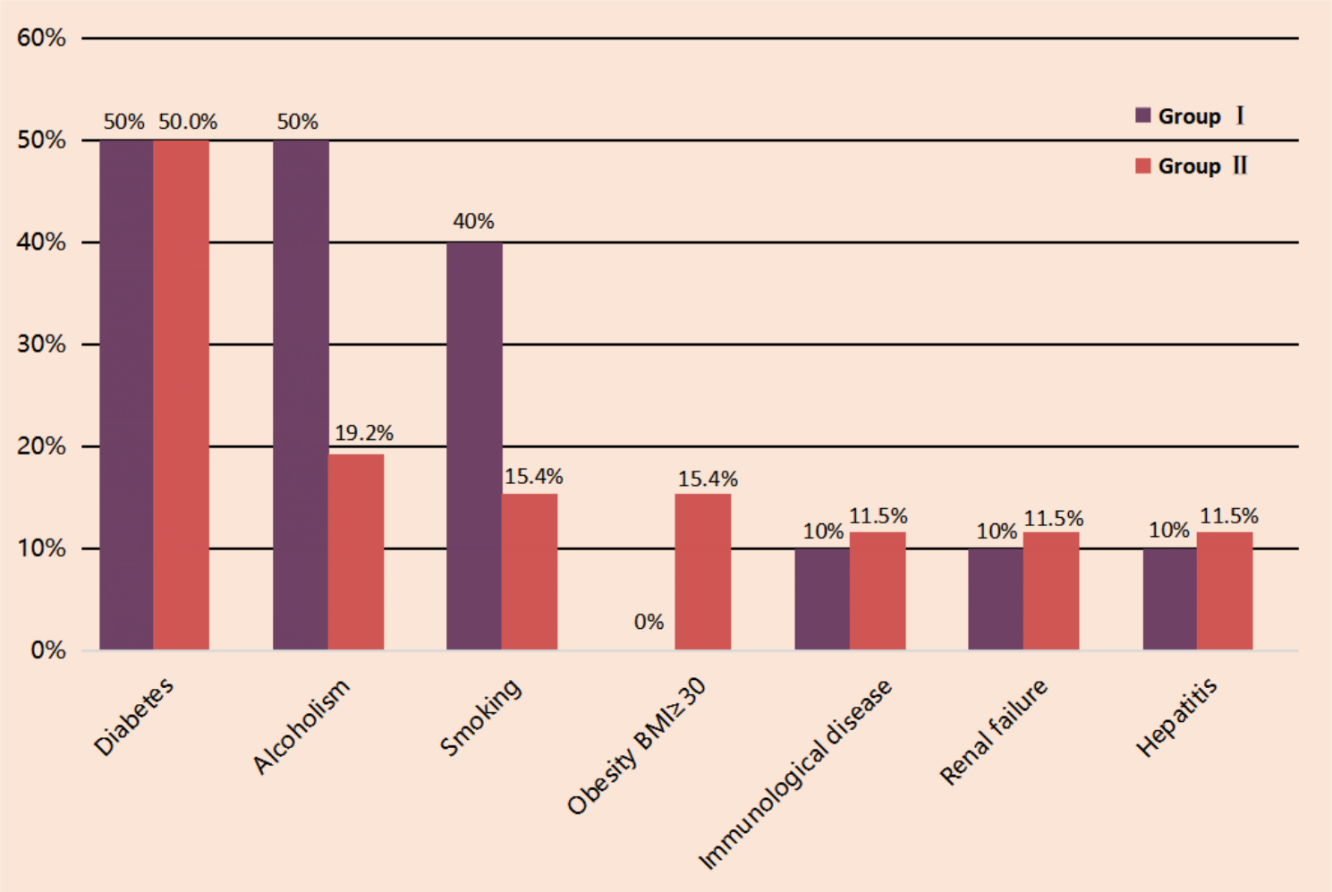
Proportions of predisposing diseases in Groups I and II

A total of 36 patients (men: 31, women: 5; mean age: 53.5 ± 11.3 [range: 28–74] years) were included in the study. All patients received surgical treatment, and there were no postoperative deaths. The predisposing diseases were as follows: diabetes mellitus: 50% (18/36), alcohol drinking: 27.8% (10/36), smoking: 22.2% (8/36), chronic renal failure: 11.1% (4/36), cirrhosis: 11.1% (4/36), obesity: 11.1% (4/36), and immunological diseases: 8.3% (3/36) (Fig. 3). Figure 4 shows the comparison of predisposing diseases in Groups I and II.

Between Groups I and II, differences in patient sex (male/female ratio: 7/3 vs. 24/2), age (53.6 ± 10.7 vs. 53.3 ± 11.7 years), BMI (22.17 vs. 24.5 kgm^− 2^), time from first debridement to wound healing (33.6 (22–60) vs. 39.6 (12–90) days) and number of debridements (2.1 [1–4] vs. 2.2 [1–5]) were not statistically significant (*P* > 0.05). An analysis of disease severity revealed the absence of significant differences in FGSI (1.5 [0–7] vs. 1.6 [0–5] points) and occurrence of shock (0 vs. 13%) between the two groups (*P* > 0.05) (Table 1; Fig. 4). However, differences in lesion diameter (15.9 (12–28) vs. 21.2 (12–40) cm) and the receipt of colostomy (0 vs. 34.6%) were statistically significant (*P* < 0.05). The two groups also differed significantly in VAS score (7.32 [5.6–9] vs. 1.94 [1.7–2.15] points), frequency of dressing changes per day (2.4 [2.16–3] vs. 0.7 [0.52–0.92] times), frequency of analgesic use per day (1.42 [0.79–2.14] vs. 0.33 [0.2–0.6] times) and LOS (19.6 [7–42] vs. 31.7 [7–84] days) (*P* < 0.05). For wound reconstruction, tension-relieving sutures for wound healing by second intention were performed in 6 patients in Group I and 24 patients in Group II, with flap transfer for wound closure conducted in 4 and 2 patients in Groups I and II, respectively. The difference in wound reconstruction method between the two groups was statistically significant (χ^2^ = 5.43, *P* = 0.04) (Table 1).

**Table 1:** Comparison of basic data and clinical outcomes of Group I and II patients

Variable	Group I	Group II	Test statistic	*P*-value
Sex, n	men: 7, women: 3	men: 24, women: 2	3.01^*^	0.12
Age (years)	53.60 ± 10.69	53.50 ± 11.70	-0.02^#^	0.99
Lesion diameter (cm)	15.90 ± 5.26	21.20 ± 7.18	-2.10^#^	0.04
FGSI (points)	1.50 ± 2.32	1.60 ± 1.36	-0.19^#^	0.85
LOS (days)	19.60 ± 12.77	31.69 ± 15.86	2.37^#^	0.028
VAS score (points)	7.32 ± 1.05	1.94 ± 0.14	16.13^#^	< 0.001
Frequency of dressing changes per day (times)	2.40 ± 0.25	0.70 ± 0.11	20.65^#^	< 0.001
Frequency of analgesic use per day (times)	1.42 ± 0.43	0.33 ± 0.1	7.89^#^	< 0.001
Time from first debridement to wound healing (days)	33.60 ± 14.35	39.60 ± 15.75	-1.04^#^	0.30
Number of debridements	2.20 ± 0.92	2.15 ± 1.16	0.11^#^	0.91
Wound reconstruction method (number of patients)	Sutures: 6, Flap: 4	Sutures: 24, Flap: 2	5.42^*^	0.04
Receipt of colostomy (number of patients)	Yes: 0, No: 10	Yes: 9, No: 17	4.61	0.04
Occurrence of shock (number of patients)	Yes: 0, No: 10	Yes: 3, No: 23	1.26^*^	0.55
Receipt of ICU treatment (number of patients)	Yes: 0, No: 10	Yes: 1, No: 25	0.40^*^	1

Note: *χ^2^ test, χ^2^ value; ^#^*t-*test, *t*-value

## Discussion

FG is a rare, severe, and rapidly progressive form of soft tissue infection with high mortality involving the perianal, perineal, and genital regions. It affects men more commonly than women [9, 10]. Diseases that weaken the immune system promote the progression of FG. Certain systemic diseases such as diabetes mellitus, chronic alcoholism, renal failure, acquired immunodeficiency syndrome (AIDS), chronic liver disease, tumors, and immunosuppression are predisposing factors for FG, [11, 12] with diabetes mellitus being the primary predisposing factor [13]. The patients included in the present study comprised 31 men (86.1%) and 5 women (13.9%) with the following predisposing diseases: diabetes mellitus (50%, 18/36), alcohol drinking (27.8%, 10/36), smoking (22.2%, 8/36), chronic renal failure (11.1%, 4/36), cirrhosis (11.1%, 4/36), obesity (11.1%, 4/36) and immunological diseases (8.3%, 3/36) (Figs. 3 and 4).

The mortality rate of FG has remained high despite the adoption of modern diagnostic and treatment techniques. Fortunately, all 36 patients in this study underwent successful surgical treatment and survived. This could possibly be ascribed to two factors: (1) Early thorough surgical debridement: The timing of surgical debridement directly affects patient outcomes. All patients included in this study underwent surgical debridement on the day of hospital admission, which contributed to more favorable outcomes; (2) Low degree of disease severity: FGSI reflects disease severity and predicts patient outcomes to a certain extent. Studies have shown that FGSI > 9 points is suggestive of severe disease commonly accompanied by septic shock and multiple organ injury. In the present study, the average FGSI of patients was 1.58 points and no patients had FGSI > 9 points. Three patients had concomitant septic shock and 1 patient required ICU treatment. Further analysis revealed that the two groups of patients had comparable FGSI scores (1.5 vs. 1.6). Despite applying different wound management methods (traditional debridement vs. VSD), the time from first debridement to wound healing was comparable between the two groups (33.6 days vs. 39.6 days). Therefore, the use of the VSD technique did not provide advantages in the general outcome and wound healing time in FG, and thorough surgical debridement remains key in FG treatment.

The three mainstays of FG treatment are fluid resuscitation, broad-spectrum anti-infective treatment and thorough debridement. However, the rapidly progressive and extensive nature of the disease often necessitates repeated debridement, thereby resulting in the enlargement of the surgical wound and difficulties in subsequent wound reconstruction. Vacuum dressing techniques (VAC and VSD), which are novel treatment methods that promote wound healing, have been widely applied in the fields of general surgery, orthopedics, burn surgery and trauma surgery [14, 15, 16]. In 2009, Cuccia et al [4]. first reported the use of VAC for FG treatment and asserted that VAC is an effective wound management technique that blocks the progression of fasciitis. Subsequent studies [3, 5−8] involving VAC in FG treatment concluded that VAC does not influence disease outcomes but enables relief of postoperative discomfort in patients and reduces physician time compared with traditional debridement. VSD, another vacuum dressing technique, is similar to VAC which involves the establishment of a closed, negative-pressure wound environment, a change from traditional active drainage to passive drainage, and the active drainage and seepage of necrotic tissue. This maintains the freshness of wounds and stimulates the growth of granulation tissue. However, certain differences exist between the two techniques: (1) VAC is mainly used for superficial wounds, whereas VSD can be applied in both superficial and deep lesion wounds; (2) VAC relies on a single suction catheter for suction while VSD involves placement of multiple VSD sponges suited to different wound sizes to provide multi-directional suction, which enables more effective drainage; (3) The drainage catheter used in VAC is not equipped with side holes, whereas the drainage catheter for VSD has side holes that allow postoperative rinsing to reduce sponge blockage and maintain unencumbered drainage.

Given the aforementioned advantages of VSD, we proposed application of VSD in wound management for patients with FG. The technique was successfully applied to treat all 26 Group II patients. Daily dressing changes were not required after VSD whereas traditional debridement and drainage required two or more dressing changes per day, which triggered the fear of pain in patients. Our results indicated that the postoperative VAS score and frequency of analgesic use in Group II were significantly lower than those in Group I, but the number of surgical debridements was not increased (2.1 vs. 2.2 days). The average LOS of patients included in this study was 28 days, which was longer than reported by Yanaral et al. and Yücel et al. [3, 17] but shorter than reported by Czymek et al [6]. We observed that the LOS of Group II patients (32 days) was significantly longer than that of Group I patients (20 days). The prolonged hospital stay of Group II may be attributed to the larger extent of lesions in Group II and the fact that VSD requires in-hospital observation. Currently, the long hospital stay associated with FG treatment is a problem that needs to be addressed [18].

The implementation of VSD requires a closed wound healing environment. However, in clinical practice, wounds in the perianal and perineal regions are usually uneven and deep with considerable amounts of discharge, and a high tendency for contamination due to the uniqueness of the anatomical structures in these regions. Consequently, the film dressing covering such wounds is easily detached and the establishment of a long-lasting and stable negative-pressure environment requires a certain level of skill. Our experiences in clinical practice are as follows: (1) Necrotic tissue that can be seen with the naked eye should be removed intraoperatively as far as possible to ensure the absence of active bleeding from the wound, as necrotic tissue and blood clots are prone to cause blockage of postoperative drainage devices, thereby resulting in drainage failure; (2) If necrosis occurs in the skin adjacent to the anal verge, we opt for laparoscopic colostomy and infuse isotonic saline from the distal end of the stoma to perform intraoperative enema, to reduce postoperative damage and contamination caused in the negative-pressure environment by stool in the intestinal tract. 25% of patients in the present study underwent laparoscopic colostomy, which is similar to the results reported by Gul et al [7].; (3) In the absence of necrosis in the skin adjacent to the anal verge, incisions are created away from the anal verge and made as small as possible. When lesions are deep and cover a large area, multiple incisions are made in the lesion area and VSD sponges of appropriate sizes are inserted through the incisions for multi-layer and multi-directional drainage, to achieve considerable reduction in the surgical wound area (Fig. 2); (4) In the case of extensive skin necrosis, thorough removal of necrotic tissue should be performed. During each dressing change for VSD, the width of the newly inserted sponge should be shorter than that of the previously inserted sponge; the sponge should be placed at the center of the wound and sutured with the peripheral healthy tissue. This will enable a gradual decrease in wound area through vacuum suction, which would be beneficial for subsequent wound reconstruction. In the present study, 8.33% (2/24) of Group II patients required wound reconstruction using flap transfer compared to 66.7% (4/6) in Group I patients, which is similar to the results reported by Zhang et al [19].

In conclusion, our findings indicate that the use of the VSD in FG treatment did not offer advantages over traditional debridement and drainage in terms of final outcome. However, compared with traditional drainage, VSD enabled a reduction in the frequency of postoperative dressing changes, enhanced pain alleviation, and decreased the frequency of analgesic use in patients with FG. VSD also enabled shrinkage of the wound area, which contributed to reduced utilization of flap transfer for wound reconstruction. Given the retrospective nature of this study, a certain degree of bias may be present in our results. In addition, the low incidence of FG has led to insufficient cases for prospective studies comparing the effects of VSD and VAC. The results of this study require further validation by multicenter prospective studies involving larger cohorts.

## Data Availability

The datasets used and analysed during the current study available from the corresponding author on reasonable request.
